# Applicability of a Green Nanocomposite Consisted of Spongin Decorated Cu_2_WO_4_(OH)_2_ and AgNPs as a High-Performance Aptasensing Platform in *Staphylococcus aureus* Detection

**DOI:** 10.3390/bios13020271

**Published:** 2023-02-14

**Authors:** Faezeh Shahdost-Fard, Shahin Faridfar, Amir Homayoun Keihan, Mohammad Aghaei, Iaroslav Petrenko, Farhad Ahmadi, Hermann Ehrlich, Mehdi Rahimi-Nasrabadi

**Affiliations:** 1 Department of Chemistry, Faculty of Basic Sciences, Farhangian University, Tehran 19396-14464, Iran; 2 Department of Chemistry, Faculty of Science, University of Imam Hossein, Tehran 15816-18711, Iran; 3 Molecular Biology Research Center, Systems Biology and Poisonings Institute, Baqiyatallah University of Medical Sciences, Tehran 19516-83759, Iran; 4 Department of Chemical Engineering, School of Chemical and Petroleum Engineering, Shiraz University, Shiraz 84334-71964, Iran; 5 Institute of Electronic and Sensor Materials, TU Bergakademie Freiberg, 09599 Freiberg, Germany; 6 Physiology Research Center, Iran University of Medical Sciences, Tehran 14496-14535, Iran; 7 Razi Drug Research Center, Iran University of Medical Sciences, Tehran 14496-14535, Iran; 8 Center for Advanced Technology, Adam Mickiewicz University, 61-614 Poznan, Poland; 9 Center of Excellence in Electrochemistry, School of Chemistry, College of Science, University of Tehran, Tehran 14179-35840, Iran

**Keywords:** spongin, cupric tungstate, silver nanoparticles, *Staphylococcus aureus*, renewable bioresource, electrochemical aptasensor

## Abstract

This study reports the synthesis of a nanocomposite consisting of spongin and its applicability in the development of an aptasensing platform with high performance. The spongin was carefully extracted from a marine sponge and decorated with copper tungsten oxide hydroxide. The resulting spongin-copper tungsten oxide hydroxide was functionalized by silver nanoparticles and utilized in electrochemical aptasensor fabrication. The nanocomposite covered on a glassy carbon electrode surface amplified the electron transfer and increased active electrochemical sites. The aptasensor was fabricated by loading of thiolated aptamer on the embedded surface via thiol-AgNPs linkage. The applicability of the aptasensor was tested in detecting the *Staphylococcus aureus* bacterium as one of the five most common causes of nosocomial infectious diseases. The aptasensor measured *S. aureus* under a linear concentration range of 10–10^8^ colony-forming units per milliliter and a limit of quantification and detection of 12 and 1 colony-forming unit per milliliter, respectively. The highly selective diagnosis of *S. aureus* in the presence of some common bacterial strains was satisfactorily evaluated. The acceptable results of the human serum analysis as the real sample may be promising in the bacteria tracking in clinical samples underlying the green chemistry principle.

## 1. Introduction

Sponges are the oldest multicellular organisms in marine and freshwater habitats. Marine spongin is held in a dense network of collagen-like microfibers [1,2,3,4]. This proteinaceous biomaterial has offered many outstanding physicochemical properties in various eco-friend and cost-effective applications [5,6,7,8]. Soft spongins have been initially considered for helmet padding, portable drinking utensils, and municipal water filters [9,10]. With the advancement of technology, their application has been extended. Their combination with carbon-based nanostructures has been drawing focused attention to various fields of applications, such as supercapacitors [11], tissue engineering [12], microbial fuel cells [13], and sensors [14,15,16]. This lightweight, flexible, conductive, and renewable source presents excellent porosity with high internal surface area [16,17], such that its metallization provides rich electrochemical active sites with catalytic properties [18]. Despite the beneficial advantages of spongin, its application in the aptasensor design has rarely been reported. Liu’s group has only reported a nanocomposite based on AuNPs/graphene sponge in the electrochemical aptasensor fabrication to measure homocysteine [19].

Different metal forms, e.g., copper (Cu) and tungsten (W), have been used as a modifier in the sensor generation and boosted various electrochemical properties [20]. It seems that utilizing copper tungsten oxide hydroxide (Cu_2_WO_4_(OH)_2_) and spongin as the beneficial low-cost modifier reduces the costs of the aptasensor fabrication process. Since the attachment of the Apt (aptamer) sequence on the surface needs a linker, the green silver nanoparticles (AgNPs) was candidate [21]. So, a novel and green nanocomposite consisting of spongin, Cu_2_WO_4_(OH)_2,_ and AgNPs, denoted as spongin-Cu_2_WO_4_(OH)_2_@AgNPs, was synthesized. Utilizing this biocompatible biomaterial in the aptasensor design may be promised some unparalleled electrochemical properties, namely (1) ensuring the fast electron transfer arising from the conductive network structure of spongin, (2) facilitating the mass transfer among the formed channels with the inherent porous structures, (3) creating rich building blocks because of the embedded available surface areas for more loading Apt sequences, and (4) guaranteed attachment of Apt sequence on the nanocomposite surface by a chemisorption linkage between the thiol group of Apt and AgNPs. In this study, the applicability of the synthesized nanocomposite has been evaluated in the aptasensor fabrication for measuring *Staphylococcus aureus* (*S. aureus*) as a target model.

Due to bacterial pathogenicity in humans, bacteria tracking is highly crucial for community health protection. *S. aureus*, an *opportunistic* pathogenic bacterium, irreparably threatens human health by causing endocarditis, toxemias of the gastrointestinal and reproductive tracts, pneumonia, and causing some invasive infections [21,22]. Since bacterial infections pose a severe threat to health systems and challenge global sustainability, developing an efficient method for pathogen footprint diagnosis in the body is constantly demanded in medical studies. Up to now, several traditional protocols have been introduced for *S. aureus* tracking based on bacterial culture and plate counting of the colony [23], polymerase chain reaction (PCR) [24,25], enzyme-linked immunosensor assay (ELISA) [26,27] and biochemical and metabolic tests [28]. Despite their accuracy, most of them make implementation challenging in clinical monitoring due to low sensitivity, interference with other similar bacterium, labor requirements, and time-consuming (3–5 days). So, the development of aptasensing methodology can be promising for the effective diagnosis of *S. aureus* because of the excellent affinity and selectivity of the Apt toward the target in less time. Although several *S. aureus* aptasensors have been reported based on various expensive nanostructures [29,30,31], utilizing the conductive and robust spongin-Cu_2_WO_4_(OH)_2_@AgNPs nanocomposite in the aptasensor design is a novel and low-cost sensing interface based on green chemistry principles. To the best of the authors’ knowledge, the nanocomposite synthesis based on green chemistry and its application in aptasensor construction has not been reported up to now.

Herein, the successful formation of the developed nanocomposite has been fully confirmed by various characterization techniques, including field emission scanning electron microscopy (FESEM), energy dispersive X-ray (EDX), X-ray diffraction (XRD), UV-vis, and Fourier-transform infrared (FTIR) spectroscopies. The aptasensor for the electrochemical trapping of *S. aureus* has been fabricated by the nanocomposite covering on a glassy carbon electrode (GCE) surface and providing a large surface area with rich AgNPs groups to load profitably Apt bioreceptors via self-assembled monolayer (SAM) linking. The *S. aureus* has been selectively measured by its incubating on the aptasensor surface and further inhibiting the electrochemical signal of the ferro/ferricyanide [Fe(CN)_6_]^3−/4−^ as the redox probe.

## 2. Experimental

### 2.1. Chemical and Reagents

*S. aureus*, Salmonella (Salmon), *Escherichia coli* (*E. coli*), and *Shigella flexneri* (*S. flexneri*) bacteria were obtained from Pasteur Institute (Tehran, Iran, https://www.pasteur.ac.ir (accessed on 8 February 2023)). A thiolated-Apt specific to *S. aureus* with a sequence of 5′-SH-TCG GCA CGT CAG TAG CGC TCG CTG GTC ATC CCA CAG CTA CGT C-3′ [32] was synthesized by Bioneer Company (Daejeon, South Korea, https://www.bioneer.com (accessed on 8 February 2023)). Ammonia (NH_3_), copper (II) sulfate (CuSO_4_), silver nitrate (AgNO_3_), ethanol, sodium hydroxide (NaOH), hydrochloride acid (HCl), potassium chloride (KCl), and sodium tungstate (Na_2_WO_4_) were ordered from Emertat Chemistry Company (Tehran, Iran, http://www.ameretatco.com (accessed on 8 February 2023)). A total of 0.1 M sterile phosphate buffer solution (PBS) with a pH of 7.4 was used for washing the enriched bacteria. For evaluating and confirming the attachment of each layer utilizing a probe sensitive to the surface state is necessary. The ferro/ferricyanide [Fe(CN)_6_]^3−/4−^, as an available and low-cost redox marker that probes the electrode surface in each step, was selected. A total of 0.1 M PBS containing 0.1 M KCl and 1 Mm [Fe(CN)_6_]^3−/4−^ as the redox marker was utilized for all the electrochemical studies. A fresh and healthy serum sample from a 31-year-old man was randomly obtained from a local clinical lab by observing ethical principles.

### 2.2. Instrumentation

Morphology characterization of nanostructures was studied by the FESEM images and EDX spectrum obtained from TESCAN (Kohoutovice, Czech Republic, https://www.tescan.com (accessed on 8 February 2023)). XRD with a Cu K_α_ radiation source was used to investigate the crystal structure of the component of the nanocomposite. An incubator was used for bacteria cultivation. A UV-vis spectrophotometer (Lambda 25, PerkinElmer, MA, USA, https://www.perkinelmer.com (accessed on 8 February 2023)) was applied for the investigation of optical density (OD) using a glass cell (1 cm) through the estimation of the cultured bacteria. FTIR spectrum was recorded by the Bruker, VERTEX 70 (Heidelberg, Germany, https://www.bruker.com (accessed on 8 February 2023)). A pH adjustment of solutions was carried out with a digital pH/mV/ion meter (Metrohm, 780 model, Herisau, Switzerland, https://www.metrohm.com/en (accessed on 8 February 2023)).

All the electrochemical experiments were performed by the potentiostat/galvanostat analyzer, including µ-Autolab III (with nova software 2.2) and FRA. A conventional three-electrode system containing a GCE with a diameter of 2 mm (Azar electrode, Urmia, Iran), Pt wire counter electrode, and Ag/AgCl (saturated KCl) reference electrode was used. The differential pulse voltammetry (DPV) technique was carried out at a pulse amplitude of 50 mV, a modulation time of 70 ms, with a scan rate of 50 mV $s^{-1}$. The electrochemical impedance spectroscopy (EIS) study was performed under a frequency range of 5 Hz–100 kHz at 0.25 V (vs. Ag/AgCl reference electrode) as a formal potential of the redox marker. Based on the result of the EIS investigation, a Randles circuit was modeled by some main components containing a constant phase element (CPE), solution ohmic resistance (R_s_), Warburg impedance (W), and charge transfer resistance (R_ct_).

### 2.3. Preparation of the Spongin

The 3D scaffold of the spongin was separated from the marine spongin and carefully washed with deionized (DI) water to remove some stone and shell parts. Next, this was immersed in 3 M HCl (two days) for pollution, eliminated and washed with DI water, and finally dried [7].

### 2.4. Spongin-Cu_2_WO_4_(OH)_2_@AgNPs Nanocomposite Synthesize

A total of 100 mg of the obtained spongin scaffold was stirred in 0.22 M Cu(NO_3_)_2_ for 24 h. Then, an aqueous solution of 15 mL Na_2_WO_4_ (0.22 M) was slightly added to it under sonication for 15 min. Next, the solution was heated at 120 °C in a stainless-steel autoclave (48 h). After washing and drying the heated mixture, 500 mg of the resulting brown powder containing the spongin-Cu_2_WO_4_(OH)_2_ was dispersed in 20 mL DI water, and 32 mg AgNO_3_ was added to them under stirring for 30 min. Then, 20 mL NaBH_4_ (13.2 mM) was droply added to them for 30 min. The resulting spongin-Cu_2_WO_4_(OH)_2_@AgNPs nanocomposite was sonicated and used for further experiments.

### 2.5. Cultivation of Bacterial Strains

*S. aureus*, Salmon, *E. coli*, and *S. flexneri* were separately cultivated in a Luria Bertani (LB) broth culture media under shaking (170 rpm, 37 °C) in an incubator (12 h). OD values of the cultured bacteria were measured by a UV-vis spectrophotometer at 600 nm. All the enriched bacteria were separately centrifuged at 6000 rpm for 20 min (25 °C), and the resulting precipitates were washed with sterile PBS (0.1 M, pH = 7.4) three times. The concentration of the bacteria was evaluated by the McFarland Turbidity Standard protocol based on a colony-forming unit per milliliter (CFU) [33]. The cultured bacteria solution was diluted by PBS under sterile conditions for further experiments.

### 2.6. Preparation of Real Samples

The human serum sample was mixed with 2% (*v/v*) ethanol and centrifuged at 5000 rpm (10 min). Then, a clear supernatant layer of the sample was diluted (10%) with 0.1 M PBS (pH = 7.4). A series of different *S. aureus* concentrations (from 1.5 × 10^3^ to 1.5 × 10^7^ CFU ${\mathrm{mL}}^{-1}$) were spiked to the pretreated sample to test the developed aptasensor.

### 2.7. Aptasensor Construction and Sensing Principle

**Scheme 1:**
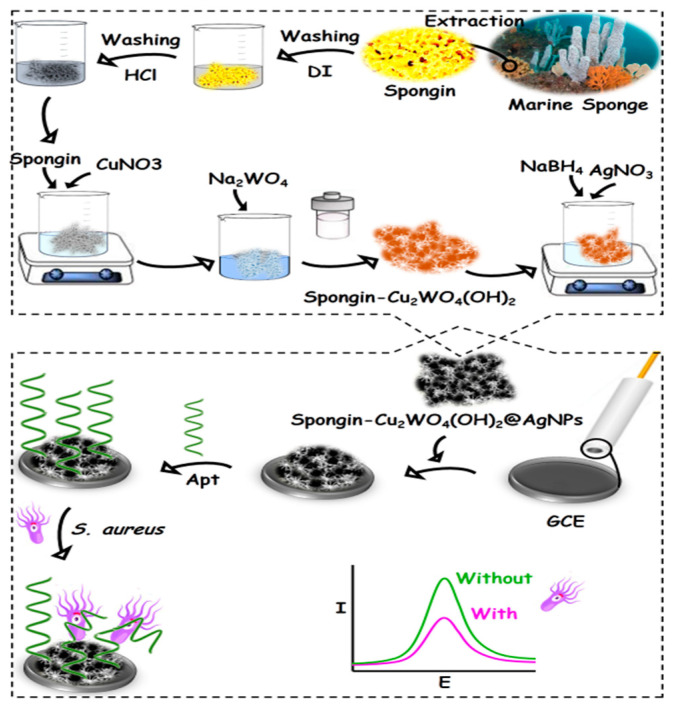
The aptasensor fabrication procedure for *S. aureus* detection.

A GCE surface was polished on an emery paper impregnated with alumina powder (0.05 µm) to create a mirror-like surface. Then, the GCE was briefly sonicated in a DI water/ethanol mixture to eliminate some possible absorbed particles. A total of 5 µL of the synthesized nanocomposite was drop-casted on the clean GCE surface and dried at room temperature to provide the spongin-Cu_2_WO_4_(OH)_2_@AgNPs/GCE. Next, 10 µL Apt was incubated on the spongin-Cu_2_WO_4_(OH)_2_@AgNPs/GCE surface overnight (4 °C) to attach to the nanocomposite’s AgNPs through SAM linkage. The resulting Apt/spongin-Cu_2_WO_4_(OH)_2_@AgNPs/GCE as the aptasensor was washed by PBS to remove some unbound particles. The signals of the aptasensor incubated by different concentrations of *S. aureus* were recorded underlying [Fe(CN)_6_]^3−/4−^ as the redox marker by the DPV technique (Scheme 1).

## 3. Results and Discussions

### 3.1. Characterization of the Spongin-Cu_2_WO_4_(OH)_2_@AgNPs Nanocomposite

**Figure 1:**
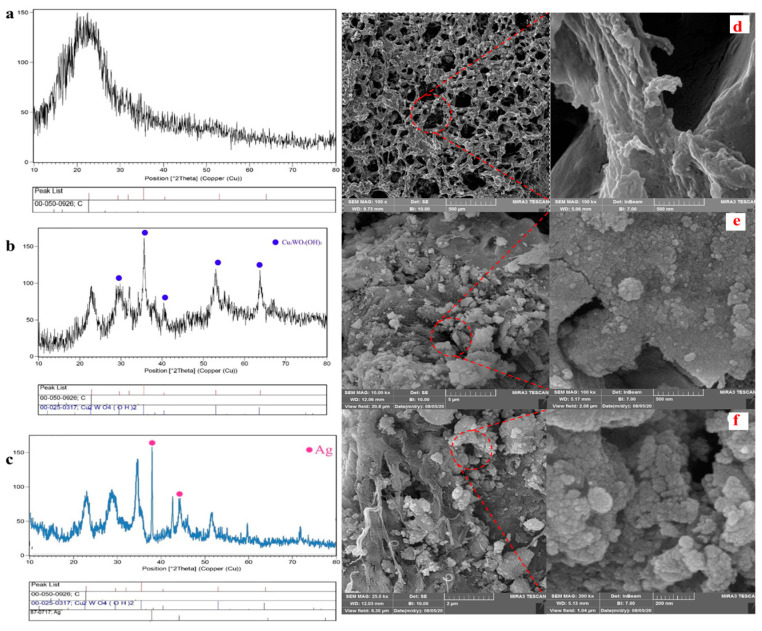
XRD patterns and FESEM images of the (**a**,**d**) spongin, (**b**,**e**) spongin-Cu_2_WO_4_(OH)_2,_ and (**c**,**f**) spongin-Cu_2_WO_4_(OH)_2_@AgNPs.

The different synthesis stages of the nanocomposite were investigated by the XRD technique. While the XRD pattern of the spongin in Figure 1a indicates a single broad peak at 2θ of 22° corroborating the carbon-based structure of the spongin, some sharp peaks marked by the blue circle confirm the presence of Cu_2_WO_4_(OH)_2_ in Figure 1b. The Ag pattern with the pink circle in Figure 1c proves a successful synthesis of the nanocomposite functionalized by AgNPs. Moreover, the absence of some common additional peaks in XRD patterns confirms the high purity of the synthesized crystalline nanocomposite. According to the Scherrer equation (Equation (1)), an average dimension (τ) of the spongin-Cu_2_WO_4_(OH)_2_ and spongin-Cu_2_WO_4_(OH)_2_@AgNPs in the nanocomposite were calculated to be 42 nm and 50 nm, respectively, where dimensionless shape factor (*K*), X-ray wavelength (*λ*), *β* as corrected full width at half-maximum after eliminating instrumental line broadening, and Bragg angle were identified.

(1) $$\tau =\frac{k\lambda }{\beta cos\theta }$$

**Figure 2:**
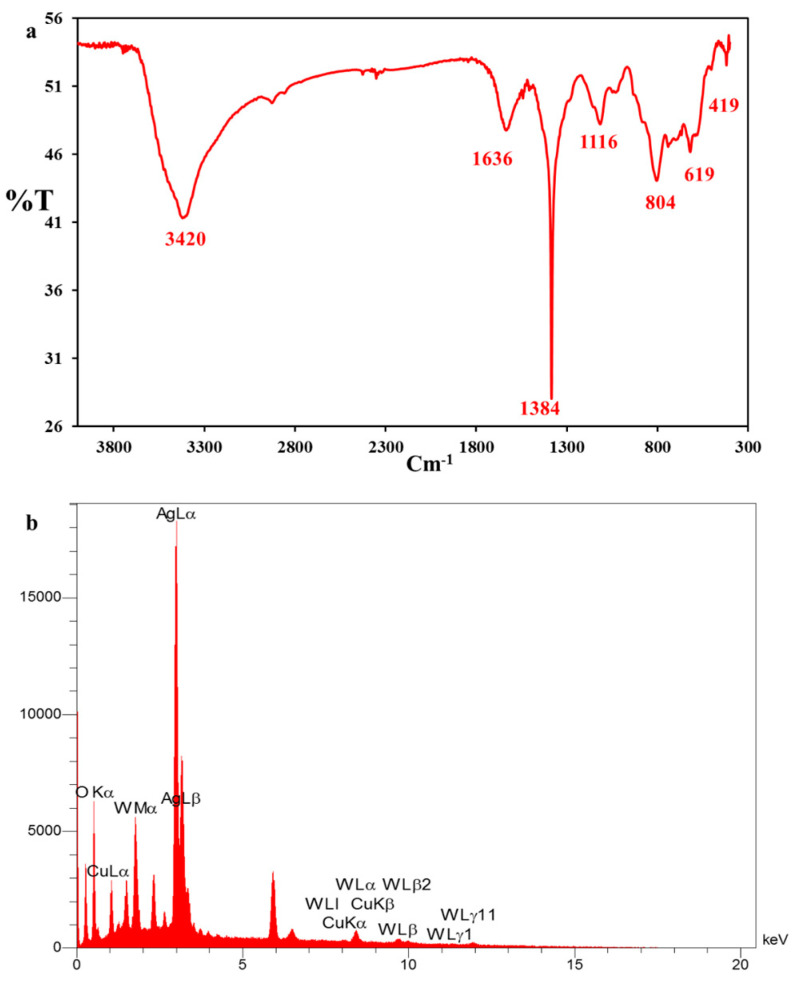
(**a**) FTIR and (**b**) EDX spectra from the spongin-Cu_2_WO_4_(OH)_2_@AgNPs.

FESEM images of the nanocomposite with two different magnifications were further recorded to identify the nanostructure (Figure 1d,f). Figure 1d exhibits a fiber network corresponding to the 3D scaffold of the spongin [34]. The morphology changes of the fiber network and the presence of some particles with different shapes and sizes in Figure 1e,f are attributed to covering Cu_2_WO_4_(OH)_2_ and Cu_2_WO_4_(OH)_2_@AgNPs on the scaffold surface, respectively. These images reveal that Cu_2_WO_4_(OH)_2_ and AgNPs are firmly attached to the spongin fibers, so they have not peeled off from the surface even during the ultrasonic washing process. FTIR spectrum of Cu_2_WO_4_(OH)_2_@AgNPs is shown in Figure 2a. The bands near 419 cm^−1^ and 619 cm^−1^ may be related to the Cu-O stretching vibration, and the peak located at 1116 cm^−1^ can be assigned to the presence of W-OH bonds [35]. The absorbance bands at 1384 cm^−1^ and 1635 cm^−1^ can be assigned to the C=O and C-O vibration modes in the carbon-based structure of the spongin, respectively. The broad band at 3420 cm^−1^ may correspond to the OH-stretching vibration modes of free and hydrogen-bonded hydroxyl groups [36]. The EDX spectrum in Figure 2b confirms the presence of all the elements of the synthesized spongin-Cu_2_WO_4_(OH)_2_@AgNPs.

### 3.2. Electrochemical Characterization of Aptasensor

**Figure 3:**
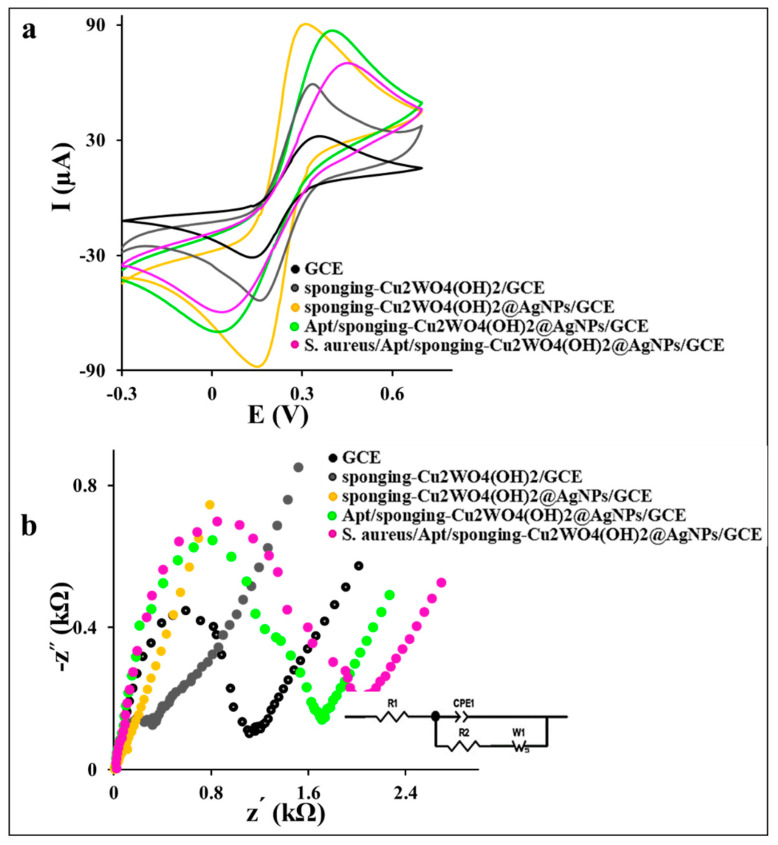
The recorded CVs (at a scan rate of 50 mV $s^{-1}$) and Nyquist curves of the GCE, spongin-Cu_2_WO_4_(OH)_2_/GCE, spongin-Cu_2_WO_4_(OH)_2_@AgNPs/GCE, Apt/spongin-Cu_2_WO_4_(OH)_2_@AgNPs/GCE, and 10^8^ CFU ${\mathrm{mL}}^{-1}$ *S. aureus* incubated on the Apt/spongin-Cu_2_WO_4_(OH)_2_@AgNPs/GCE in 0.1 M PBS containing 0.1 M KCl and 1 mM [Fe(CN)_6_]^3−/4−^ as the redox marker.

The GCE modification process was investigated step by step with the recording of the corresponding cyclic voltammograms (CVs) of GCE in 0.1 M PBS containing 0.1 M KCl and 1 mM [Fe(CN)_6_]^3−/4−^ as the redox marker at a scan rate of 50 mV $s^{-1}$ (Figure 3a). As shown in Figure 3a, the CV of the spongin-Cu_2_WO_4_(OH)_2_/GCE exhibits a higher current signal with a lower peak-to-peak potential separation (ΔE = 145 mV) compared to the bare GCE (ΔE = 165 mV). This desired electrochemical behavior reveals the efficient ability of the spongin-Cu_2_WO_4_(OH)_2_ in activating the GCE sites and amplifying the electron transfer. The higher current signal and lower ΔE value (142 mV) of spongin-Cu_2_WO_4_(OH)_2_@AgNPs/GCE than the spongin-Cu_2_WO_4_(OH)_2_/GCE is attributed to the AgNPs presence in the nanocomposite. The AgNPs not only increase the surface conductivity and enhance the current signal than spongin-Cu_2_WO_4_(OH)_2_/GCE and bare GCE but also act as a linker for the thiolated-Apt attachment on the nanocomposite via chemisorption linkage. The current signal decrease and ΔE value increase (388 mV) in the Apt/spongin-Cu_2_WO_4_(OH)_2_@AgNPs/GCE layer is assigned to the repelling of the negatively charged Apt attached to the AgNPs and the anion [Fe(CN)_6_]^3−/4−^ probe. This behavior confirms the attachment of thiolated-Apt to the AgNPs on the nanocomposite surface. The *S. aureus*/Apt complex formation on the surface is confirmed by further electron transfer decrease and the ΔE value increase (416 mV) stemming from the *S. aureus* attachment on the Apt’s arm and more space barrier created on the surface.

The electron transfers kinetic study of the aptasensor’s different layers by the EIS technique and corresponding Nyquist plots in Figure 3b exhibits a large semicircle with a charge transfer resistance (R_ct_) value of 1.089 kΩ and 0.289 kΩ for the bare GCE and spongin-Cu_2_WO_4_(OH)_2_/GCE, respectively. This behavior can be attributed to the high surface area and porosity of the spongin-Cu_2_WO_4_(OH)_2_ than the GCE to improve electron transfer and mass transfer on the surface. A low R_ct_ value (0.03 kΩ) with a straight line for the spongin-Cu_2_WO_4_(OH)_2_@AgNPs/GCE layer proves the AgNPs presence in the nanocomposite. A remarkable increase in the R_ct_ value (1.692 kΩ) for the Apt/spongin-Cu_2_WO_4_(OH)_2_@AgNPs/GCE is attributed to the immobilization of the negatively charged Apt on the GCE surface via AgNPs linker. More increase in R_ct_ value to 2.018 kΩ is assigned to *S. aureus* capturing by the Apt and suitable performance of the aptasensor in the target tracking. The CV and EIS results certify the successful immobilization of all layers in the aptasensor fabrication process.

To evaluate the performance of the embedded nanocomposite in the surface area increasing, the electroactive surface area (*A*) values of GCE, spongin-Cu_2_WO_4_(OH)_2_/GCE, and spongin-Cu_2_WO_4_(OH)_2_@AgNPs/GCE surfaces were investigated by using the famous Randles–Sevcik Equation (2) [37]:

*I_p_* = (2.69 × 10^5^) *n*^3/2^ *A D*^1/2^ *C ν*^1/2^ (2)

where *D* is the diffusion coefficient of K_3_[Fe(CN)_6_] (7.6 × $10^{-6}$ cm^2^ $s^{-1}$) and all symbols have a normal meaning. Accordingly, the A values of the GCE, spongin-Cu_2_WO_4_(OH)_2_/GCE, and spongin-Cu_2_WO_4_(OH)_2_@AgNPs/GCE surfaces were calculated to be 0.03, 0.06, and 0.08 cm^2^, respectively. The estimated A value of 2.67-fold for the spongin-Cu_2_WO_4_(OH)_2_@AgNPs/GCE surface confirms the unique properties of the proposed nanocomposite in supplying higher surface area and amplifying the current signal.

### 3.3. Optimization of Incubation Time

**Figure 4:**
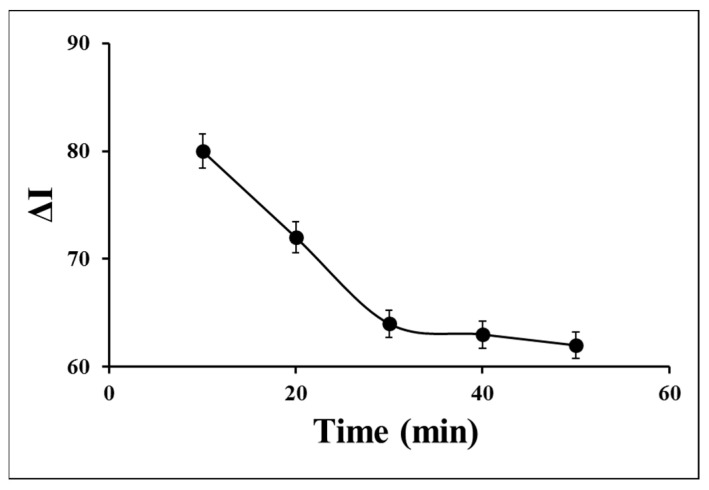
Effect of incubation time of *S. aureus* on the aptasensor signal for 1.5 × 10^6^ CFU ${\mathrm{mL}}^{-1}$ of *S. aureus* in 0.1 M PBS containing 0.1 M KCl and 1 mM ferro/ferricyanide $\left({\left[\mathrm{Fe}{\left(\mathrm{CN}\right)}_{6}\right]}^{3-/4-}\right)$ as a redox marker.

The incubation time strongly influences the response stability of the aptasensor. To appraise the incubation time of *S. aureus* on the aptasensor surface, different times from 10 min to 50 min were investigated by monitoring the recorded DPVs in 0.1 M PBS containing 0.1 M KCl and 1 mM [Fe(CN)_6_]^3−/4−^ (Figure 4). The current difference (ΔI) value in the absence and presence of 1.5 × 10^6^ CFU ${\mathrm{mL}}^{-1}$ of *S. aureus* was significantly decreased by the time increasing from 10 min to 30 min, attributing to occupying the surface’s active sites via the target and the target-Apt complex forming on the surface. Since the ΔI value was not approximately changed by more times from 30 min to 50 min and finally tended to a steady state, 30 min was considered the optimum incubation time for further experiments.

### 3.4. Analytical Performance of the Aptasensor

**Figure 5:**
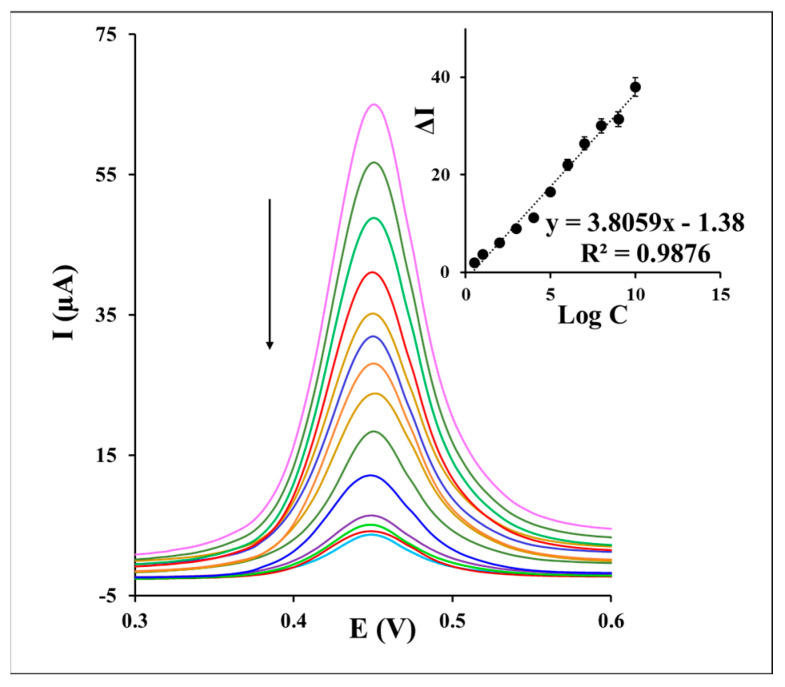
The DPVs of the aptasensor and inset a corresponding calibration curve after incubation with a series of different concentrations of *S. aureus* from 10 CFU ${\mathrm{mL}}^{-1}$ to 10^8^ CFU ${\mathrm{mL}}^{-1}$ during an incubation time of 30 min in 0.1 M PBS containing 0.1 M KCl and 1 mM ${\left[\mathrm{Fe}{\left(\mathrm{CN}\right)}_{6}\right]}^{3-/4-}$ as the redox marker.

To appraise the analytical performance of the aptasensor, a series of different concentrations of *S. aureus* was injected into the aptasensor for 30 min. As indicated in Figure 5, the recorded DPVs of the aptasensor in the redox probe solution displays a current decrease by the increasing of the *S. aureus* concentration from 10 CFU ${\mathrm{mL}}^{-1}$ to 10^8^ CFU ${\mathrm{mL}}^{-1}$. This behavior may be stemmed from the fact that more concentrations of *S. aureus* on the aptasensor surface form more of the *S. aureus*/Apt complexes and enhance the electron transfer hindrance of redox marker on the sensing interface. Inset of Figure 5 presents a regression dependence of the current response to the logarithm of different *S. aureus* concentrations, as the analytical calibration depicted under an equation of ∆I (µA) = 3.8059 log C*_S. aureus_* − 1.38 (R^2^ = 0.9876). Accordingly, a limit of quantification (LOQ) of 12 CFU ${\mathrm{mL}}^{-1}$ and detection (LOD) of 1 CFU ${\mathrm{mL}}^{-1}$ were calculated based on the S/N of 3. The examination of the *S. aureus* assay (1.5 × 10^3^ CFU ${\mathrm{mL}}^{-1}$) by the aptasensor through five times repetitions demonstrated suitable repeatability of the method with a relative standard deviation (RSD%) value of 3.52%. Furthermore, the detection of 1.5 × 10^3^ CFU ${\mathrm{mL}}^{-1}$ of *S. aureus* by the five fabricated aptasensors confirmed a reproducible response of the aptasensor under the RSD% value of 2.59%.

**Figure 6:**
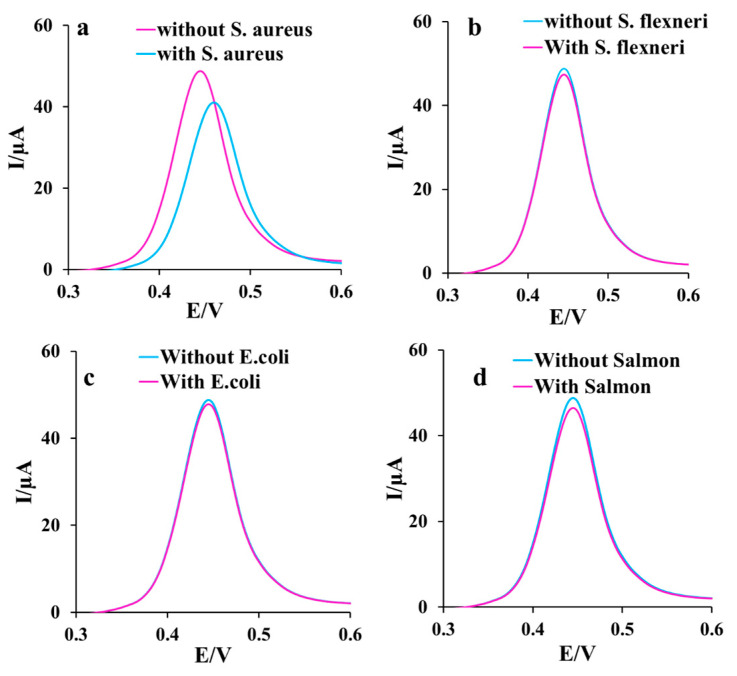
The DPVs of the aptasensor after incubation of 1.5 × 10^7^ CFU ${\mathrm{mL}}^{-1}$ of (**a**) *S. aureus*, (**b**) *S. flexneri*, (**c**) *E. coli*, and (**d**) Salmon for 30 min in 0.1 M PBS containing 0.1 M KCl and 1 mM ([Fe(CN)_6_]^3−/4−^) as the redox marker.

Anti-interference ability of the aptasensor in the *S. aureus* monitoring in the presence of other similar bacteria is crucial in avoiding false identification. To appraise the selectivity of the aptasensor, three different strains of the cultured bacteria, including Salmon, *E. coli*, *S. flexneri*, and *S. aureus* (1.5 × 10^7^ CFU ${\mathrm{mL}}^{-1}$), were separately measured (Figure 6a–d). *S. aureus* remarkably changed the signal, while other bacteria strains had no significant interference in the aptasensor response in 0.1 M PBS containing 0.1 M KCl and 1 mM [Fe(CN)_6_]^3−/4−^. So, the specific analysis of the aptasensor is confirmed. This satisfactory performance of the developed aptasensor compared to some reported sensors and aptasensors listed in Table 1 can be asserted due to using the efficient sensing interface because of some reasons as follows: (1) facilitating the mass transfer among the formed channels of the spongin with the porous network, (2) enhancing the electron transfer among the spongin-Cu_2_WO_4_(OH)_2_ conductive structure, (3) embedding a high surface to volume ratio in the presence of AgNPs, (4) providing more loading of Apt sequences on the surface via a strong bonding through SAM linkage, and (5) amplifying the sensing selectivity by utilizing Apt.

### 3.5. Real Sample Analysis

The human serum as the real sample was tested to examine the feasibility of the aptasensor for *S. aureus* tracking. The DPVs of the pretreated serum samples (described in the experimental part) analyzed by the aptasensor were recorded, and the results were evaluated. A series of calculated recovery values in the range of 94.2% to 103.4% with an RSD% value less than 3.01% revealed acceptable screening of *S. aureus* in the serum sample by the developed aptasensor.

## 4. Conclusions

To sum up, the highly porous spongin-Cu_2_WO_4_(OH)_2_@AgNPs nanocomposite was greenly synthesized. This biocompatible nanocomposite was utilized as the GCE modifier in the aptasensor fabrication due to its fast mass and electron transfer with a large surface area. The highly sensitive sensing interface with many active sites was achieved by loading Apt captures on the nanocomposite via AgNPs linkage. By binding *S. aureus* on the aptasensor surface and the corresponding increasing spatial barrier of the surface, the *S. aureus* was sensitively measured under a wider LDR with lower LOD, and LOQ values than many previously reported aptasensors. *S. aureus* was selectively distinguished in the presence of some similar strains of the cultured bacteria and human serum. The proposed methodology is promising for the nanocomposite immobilization on other carbon-based platforms as the electrode, such as carbon cloth, felt graphite, or screen-printed carbon electrode (SPCE), to attach further layers of AgNPs and Apt. Accordingly, a flexible and miniaturized aptasensor based on flexible or flat surfaces can be generated for *S. aureus* detection. Furthermore, various aptasensors can be designed for sensing other targets by only changing the Apt sequence. Because the reticular structure of the spongin gives adequate access to the catalysis process, this renewable bioresource can be used for more electrochemical fields, such as supercapacitors, batteries, and fuel cells.

## Figures and Tables

**Table 1:** A comparison of the analytical performance of the aptasensor with some reported sensors and aptasensors in *S. aureus* detection.

Strategy	Technique	LOD CFU mL^−1^	LDR CFU mL^−1^	Ref.
Apt/*S. aureus*/Apt-AgNP	DPV	1	10–10^6^	[38]
Apt/rGO/AuNPs	Impedimetric	10	10–10^6^	[39]
Apt/gold electrode	Impedimetric	10	10–10^4^	[40]
Apt/AuNPs/CNPs/CNFs	Impedimetric		1.2 × 10^1^–1.2 × 10^8^	[29]
Apt/SWCNT	Potentiometric		10^3^–10^8^	[41]
Apt/AuNPs	Colorimetric	9	10–10^6^	[42]
CNT/Apt	Potentiometric	8 × 10^2^	8 × 10^2^–10^8^	[41]
Gold-based immunosensors	PM-RAIRS	10^5^	–	[43]
CdSe QD	Fluorometric	10^2^	10^2^–10^7^	[44]
Apt/spongin-Cu_2_WO_4_(OH)_2_@AgNPs/GCE	DPV	1	10–10^8^	This study

rGO: reduced graphene oxide. SWCNT: single-walled carbon nanotube. CNP: carbon nanoparticles. CNF: cellulose nanofibers. CNT: carbon nanotube. PM-RAIRS: polarization modulation reflection absorption infrared spectroscopy. QDs: quantum dots.

## Data Availability

Not applicable.
